# Trps1 Regulates Development of Craniofacial Skeleton and Is Required for the Initiation of Palatal Shelves Fusion

**DOI:** 10.3389/fphys.2019.00513

**Published:** 2019-05-03

**Authors:** Kah Yan Cho, Brian P. Kelley, Daisy Monier, Brendan Lee, Heather Szabo-Rogers, Dobrawa Napierala

**Affiliations:** ^1^ Department of Orthodontics and Dentofacial Orthopedics, School of Dental Medicine, University of Pittsburgh, Pittsburgh, PA, United States; ^2^ Department of Molecular and Human Genetics, Baylor College of Medicine, Houston, TX, United States; ^3^ Section of Plastic and Reconstructive Surgery, Department of Surgery, University of Michigan, Ann Arbor, MI, United States; ^4^ Department of Oral Biology, School of Dental Medicine, University of Pittsburgh, Pittsburgh, PA, United States; ^5^ Department of Developmental Biology, School of Medicine, University of Pittsburgh, Pittsburgh, PA, United States; ^6^ McGowan Institute for Regenerative Medicine, University of Pittsburgh, Pittsburgh, PA, United States

**Keywords:** TRPS1, trichorhinophalangeal syndrome, palatal development, craniofacial, palatal fusion, secondary palate

## Abstract

Trichorhinophalangeal syndrome (TRPS) is an autosomal dominant disorder resulting from heterozygous mutations of the *TRPS1* gene. Common craniofacial abnormalities in TRPS patients include micrognathia, hypoplastic zygomatic arch, high-arched palate, and, occasionally, cleft palate. Studies have demonstrated that mice with a heterozygous *Trps1* mutation (*Trps1^+/−^* mice) have similar features to patients with TRPS, including high-arched palates. However, mice with a homozygous *Trps1* mutation (*Trps1^−/−^* mice) exhibit similar but more severe abnormalities, including cleft palate. Our study aimed to characterize the craniofacial phenotype to understand the role of *Trps1* in craniofacial development and gain insight on the cleft palate pathogenesis in *Trps1* deficiency. Whole-mount skeletal staining revealed hypoplastic skeletal and cartilaginous elements, steep nasal slope, and missing presphenoid in *Trps1^−/−^* mice. Although several craniofacial skeleton elements were abnormal in *Trps1^−/−^* mice, the *Trps1* deficiency did not appear to disrupt cranial vault development. All *Trps1^−/−^* mice presented with cleft palate. Analyses of *Trps1* expression during palatogenesis detected Trps1 mRNA and protein in palatal mesenchyme and in specific regions of palatal epithelium, which suggested that Trps1 is involved in palatal fusion. *Ex vivo* culture experiments demonstrated that *Trps1^−/−^* palatal shelves were unable to initiate the fusion process. On the molecular level, Trps1 deficiency resulted in decreased epithelial expression of proteins involved in palatal fusion, including chondroitin sulfate proteoglycan, transforming growth factor-beta 3, Twist1, and beta-catenin. Mesenchymal expression of chondroitin sulfate proteoglycan expression was unaffected, indicating a cell type-specific mechanism of *Trps1* regulation on chondroitin sulfate proteoglycan. In conclusion, we demonstrated that *Trps1* is involved in the development of craniofacial skeletal elements and in the initiation of the palatal shelves fusion. Furthermore, our studies uncovered that Trps1 is required for epithelial expression of several proteins involved in the palatal shelves fusion.

## Introduction

Orofacial clefting, including cleft lip/palate (CLP), is one of the most common birth defects in the United States, occurring in approximately 1/700 live births with around 4,440 new cases occurring every year (Parker et al., 2010; Dixon et al., 2011). The management of CLP requires multiple surgeries to establish proper function and esthetics, which can be a significant burden to patients and their families. CLP may present as an isolated feature or may be associated with conditions such as Pierre Robin and Treacher Collins (Venkatesh, 2009). These syndromes also frequently present with skeletal malformations including retrognathia, midface hypoplasia, and temporomandibular joint (TMJ) abnormalities (Chang and Steinbacher, 2012; Gangopadhyay et al., 2012). Thus, the pathogenesis of CLP may be related to a dysregulation of genes involved in skeletal development.

In order to study the pathogenesis of cleft palate, we must first understand the process of palatogenesis. Palatogenesis requires cross-communication between multiple signaling pathways to orchestrate molecular events at each step of palatal development. Palatogenesis begins with palatal processes growing vertically on each side of the tongue, then elevating horizontally above the tongue. The opposing palatal shelves must approximate each other at the midline and adhere together *via* cell adhesion molecules to form the midline epithelial seam (MES), which subsequently disintegrates during palatal shelf fusion. Various mechanisms have been implicated in the palatal shelves fusion, including epithelial cell apoptosis, extrusion, migration or transition to the mesenchymal state *via* the epithelial-to-mesenchyme transition (EMT) process (Bush and Jiang, 2012). An interruption at any stage of this complex process can lead to cleft palate.

Cleft palate has been reported in rare cases of the trichorhinophalangeal syndrome (TRPS) (Morioka et al., 1999; Solc et al., 2017), an autosomal dominant disorder resulting from mutations in the *TRPS1* gene, which encodes the transcriptional repressor TRPS1. Characteristic clinical features of TRPS include sparse hair, bulbous nose, micrognathia, cone-shaped epiphyses of phalanges, and dental abnormalities (Giedion, 1966; Bennett et al., 1981; Ludecke et al., 2001; Kantaputra et al., 2008). A mouse model of TRPS with a heterozygous mutation of the *Trps1* gene (*Trps1^+/−^* mice) demonstrates subtle craniofacial malformations such as abnormal palatal arch, shortened mandible, and abnormal zygomatic arch (Malik et al., 2002). However, in mice with a homozygous mutation of *Trps1* (*Trps1^−/−^* mice), cleft palate was observed, suggesting a dose-dependent effect of *Trps1* deficiency on palate formation (Kantaputra et al., 2008).

Although the craniofacial phenotype of TRPS patients clearly indicates that *TRPS1* is involved in palatogenesis and craniofacial development, the specific role of *TRPS1* in these processes and mechanisms underlying the craniofacial abnormalities in TRPS are unknown. To address this gap in our knowledge, we analyzed *Trps1^−/−^* mice and focused on uncovering molecular and cellular mechanisms leading to the cleft palate in *Trps1* deficiency. Considering the suggested role of *Trps1* in EMT in kidney, liver, and during tumorigenesis (Gai et al., 2009; Su et al., 2014; Zhe et al., 2015), we focused our studies on the palatal shelf fusion.

## Materials and Methods

### Mice

*Trps1^−/−^* and *Trps1^+/−^* mice were described previously (Malik et al., 2002), and maintained on 129svev and C57BL/6J backgrounds. For timed matings, the day the plug was observed was designated E0.5. All animal work was carried out under approved IACUC protocols.

### Whole-Mount Skeletal Staining

Skeletal staining of E18.5 C57BL/6J wild type (WT) (*n* = 5) and *Trps1^−/−^* (*n* = 4) mouse embryos was performed with 0.03% Alcian blue (*Sigma-Aldrich, A3157*) and 0.01% Alizarin red (*Sigma- Aldrich, A5533*) solution. Imaging was performed on Leica M165FC microscope and Leica Application Suite software.

### *Ex vivo* Palatal Shelf Culture

Palatal shelves from E13.5 129svev WT (*n* = 11), *Trps1^+/−^* (*n* = 5), and *Trps1^−/−^* (*n* = 11) mice were dissected. Pairs of palatal shelves were placed on trans-well inserts (8 μm pore size) and incubated in α-MEM (without serum) at 37°C for 48 h as described in Wang et al. (2013).

### RNA *in situ* Hybridization

Embryos were fixed with 4% paraformaldehyde, dehydrated, and embedded in paraffin following standard protocols. *In situ* hybridization was performed as described previously (Morello et al., 2001; Napierala et al., 2008).

### H&E Staining

Seven-micrometer-thick sections of paraffin-embedded 129svev mice and palatal shelves were stained with H&E according to standard protocols. The stained tissues were mounted in mounting medium (*Richard-Allan Scientific*).

### Fluorescent Immunohistochemistry

C57BL/6J WT and *Trps1^−/−^* mice were fixed in either 4% paraformaldehyde (for detection of Trps1 and Tgfβ3) or Carnoy’s solution (for detection of chondroitin sulfate proteoglycan (CSPG), β-catenin, and Twist1) and embedded in paraffin. Heat-induced epitope retrieval was performed in sodium citrate buffer (10 mM sodium citrate, 0.05% Tween20, pH = 6.5). The primary antibodies used were 1:50 rabbit anti-TRPS1 (*Abnova, PAB17465*), 1:200 anti-chondroitin sulfate proteoglycan (*Sigma, C8035*), 1:100 rabbit anti-TGFβ3 (*Abcam, ab15537*), 1:250 rabbit anti-β-catenin (*Abcam, ab50581*), 1:100 rabbit anti-Twist1 (*Abcam, ab32572*), and 1:20 anti-Ki67 (*Santa Cruz, sc-7846*). Anti-rabbit AlexaFlour 488-conjugated secondary antibody (*Thermo Fisher Scientific*) and ProLong Gold with DAPI Antifade Mountant (*Molecular Probes, Thermo Fisher Scientific*) were used. Images were taken with Zeiss AxioCam on a Zeiss Axioskop A1 microscope and ZEN software.

### Apoptosis Assay

Apoptosis was evaluated using TUNEL assay (*Thermo Fisher Scientific*) according to the manufacturer’s protocol.

### Measurements and Statistical Analysis

Total head length was measured from the tip of nasal cartilage to supraoccipital bone. The nasal length was measured from the tip of nasal cartilage to the frontonasal suture. The nasal angle was taken between the plane connecting the tip of the nasal cartilage to the superior border of supraoccipital bone and the plane tangent to the nasal bone from the images using a protractor. Mandible length was measured from the most posterior point at the condylar process to the most anterior point at the symphysis. Lengths of the head, nose, and mandible were measured with calipers. Nonparametric Wilcoxon rank sum test was used to determine a difference between WT and *Trps1^−/−^* mice head, nose, and mandible measurements with *α* = 0.05. The statistical analysis was performed using StataSE software.

## Results

### Craniofacial Characteristics of *Trps1^−/−^* Mice Is a Phenocopy of the Clinical Presentation of TRPS Patients

**Figure 1:**
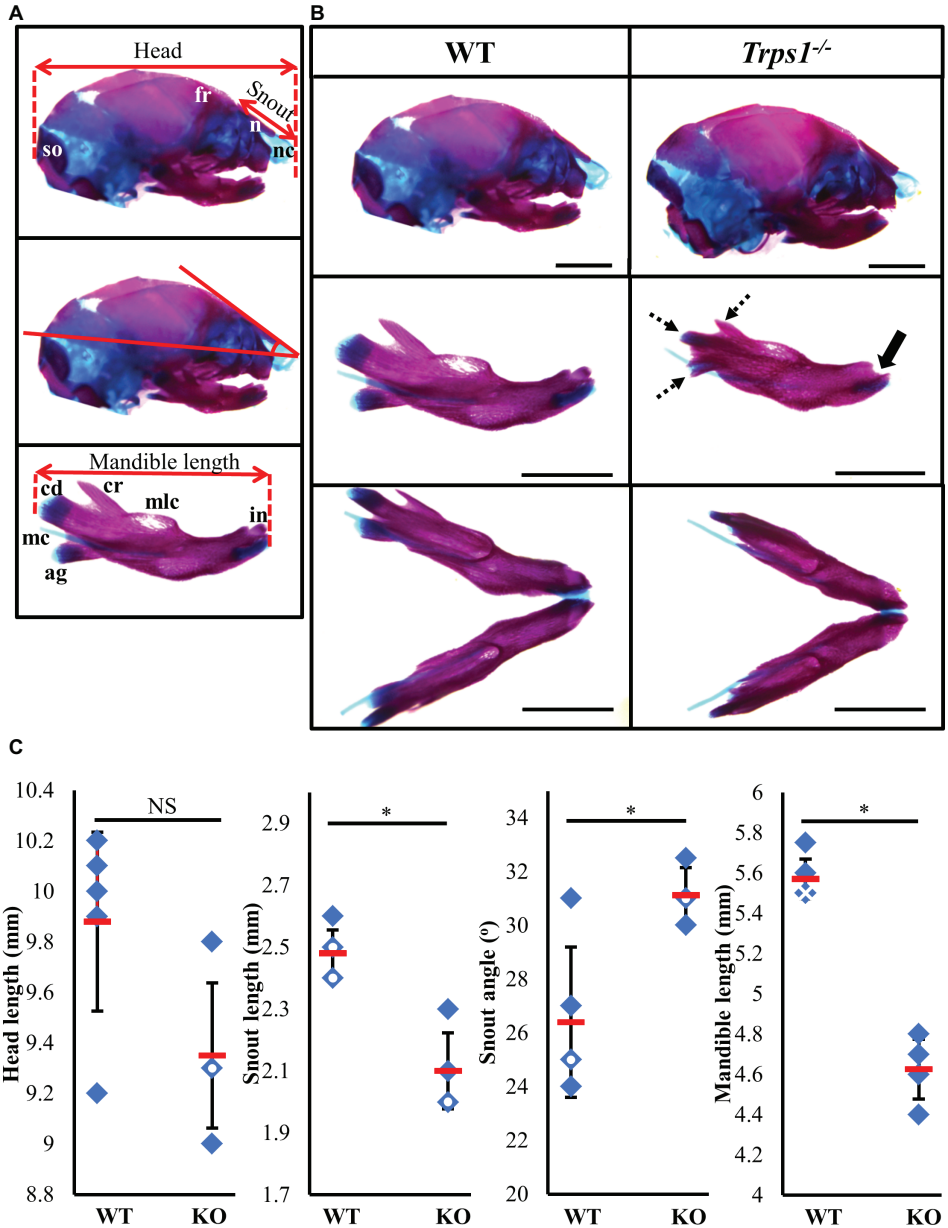
Craniofacial skeleton abnormalities of *Trps1^−/−^* mice compared to WT. Measurements were done on E18.5 head skeletal preparations stained with Alcian blue and Alizarin red. Representative images are shown. **(A)** Landmarks used for head measurements. **(B)** Comparison of E18.5 WT (left) and *Trps1^−/−^* (right) lateral skull (top panel) and mandible (middle panel: lateral view; bottom panel: intraoral view). Coronoid process, condylar cartilage, and angular cartilage of *Trps1^−/−^* mice were hypoplastic (dotted arrows). The mandibular incisors have not erupted in *Trps1^−/−^* mandible (solid arrow). Meckel’s cartilage and molar crypts were present in both WT and knockout phenotypes. **(C)** There was no significant difference in total length of the head between the two groups but the nose was significantly shorter and downward-sloping in the *Trps1^−/−^* mice. The *Trps1^−/−^* mandible was also significantly shorter than WT. Red dashes represent the mean value in each data set. Solid data points represent individual samples, data points with white circle within indicate two samples with the same measurement and data points with white cross within indicate three samples with the same measurement. Error bars indicate the standard deviation of the sample. Asterisk indicates statistically significant differences between the WT and *Trps1^−/−^* groups (*p* < 0.05). *Scale bar: 2 mm. ag* – *angular process, cr* – *coronoid process, cd* – *condylar process, fr* – *frontal bone, in* – *incisor, mc* – *Meckel’s cartilage, mlc* – *molar crypt, n* – *nasal bone, nc* – *nasal cartilage, so* – *supraoccipital.*

*TRPS1* haploinsufficiency in humans results in a characteristic facial appearance, which suggests a disturbed development of the skeletal elements. This, in addition to the known role of *Trps1* in appendicular skeleton development, led us to studies addressing the role of *Trps1* in the formation of the craniofacial skeleton. *Trps1^+/−^* mice have a mild craniofacial phenotype, while *Trps1^−/−^* mice die at birth (Malik et al., 2002). Due to the neonatal lethality of *Trps1^−/−^* mice, we focused our analyses on E18.5 embryos. Comparisons of skeletal preparations of E18.5 WT and *Trps1^−/−^* mice showed that the mean head length of *Trps1^−/−^* head (9.4 ± 0.3 mm) was not significantly different from WT (9.9 ± 0.4 mm) (Figure 1). However, the mean nasal length was significantly shorter in *Trps1^−/−^* (2.1 ± 0.1 mm) than the WT littermate controls (2.5 ± 0.1 mm) (*p* < 0.05) (Figure 1). The mean nasal angle of the *Trps1^−/−^* mice (31.1 ± 1^°^) was also significantly steeper than the WT (26.4 ± 2.8^°^) (*p* < 0.05) (Figure 1). No apparent differences in the nasal cartilage were detected.

**Figure 2:**
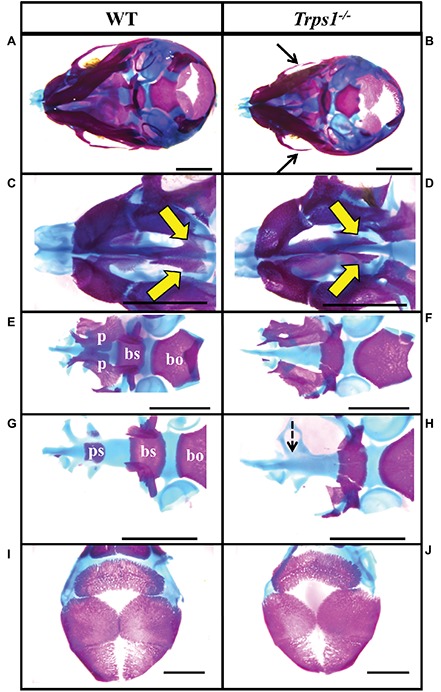
Zygomatic arch and cranial base abnormalities in *Trps1^−/−^* mice. Skeletal staining of E18.5 WT (left) and *Trps1^−/−^* (right) mice. **(A,B)** Inferior view of skull demonstrating hypoplastic zygomatic process (black arrows) of the maxillary bone in *Trps1^−/−^* mice. **(C,D)** Magnified view of the nasal area illustrating underdeveloped vomer bones in *Trps1^−/−^* mice (yellow arrows). **(E,F)** The basioccipital bone is unaffected in *Trps1^−/−^* mice, while the basisphenoid bone is smaller and the cleft palate is evident with the unfused palatal shelves. **(G,H)** Removal of palatine bones revealed the absence of presphenoid bone in *Trps1^−/−^* mice (dotted arrow). **(I,J)** Cranial vault bones showed no apparent differences between the two groups. *Scale bar: 2 mm. bo* – *basioccipital, bs* – *basisphenoid, p* – *palatine bone, ps* – *presphenoid.*

Multiple differences between WT and *Trps1^−/−^* mandibles were also detected. The *Trps1^−/−^* mutant phenotype includes unerupted incisors, reduced mineralized regions as well as less cartilage in the coronoid, condylar, and angular processes (Figure 1B). *Trps1^−/−^* mandibles (4.6 ± 0.1 mm) were significantly shorter than the WT counterparts (5.6 ± 0.1 mm) (*p* < 0.05) (Figure 1). Meckel’s cartilage and clearly demarcated molar crypts were present in both genotypes (Figure 1). Zygomatic arches were less developed in the *Trps1^−/−^* mice in comparison with WT littermates (Figures 2A,B), which is consistent with the craniofacial skeletal phenotype of adult *Trps1^+/−^* mice (Malik et al., 2002). This reduction in mandibular and midfacial structures resembles the jaw hypoplasia observed in patients with TRPS. Additionally, analysis of the skeletal preparations revealed underdeveloped vomer bones in *Trps1^−/−^* mice (Figures 2C,D). Finally, we observed cranial base abnormalities in *Trps1^−/−^* mice, where the basisphenoid was smaller and the presphenoid bone was absent (Figures 2E–H). However, there was no difference in the basioccipital and cranial vault bones between the two genotypes (Figures 2I,J). These results suggest that *Trps1* is important specifically for the development of the nose, jaw, and cranial base.

### Trps1 Deficiency Results in Cleft Palate Phenotype

**Figure 3:**
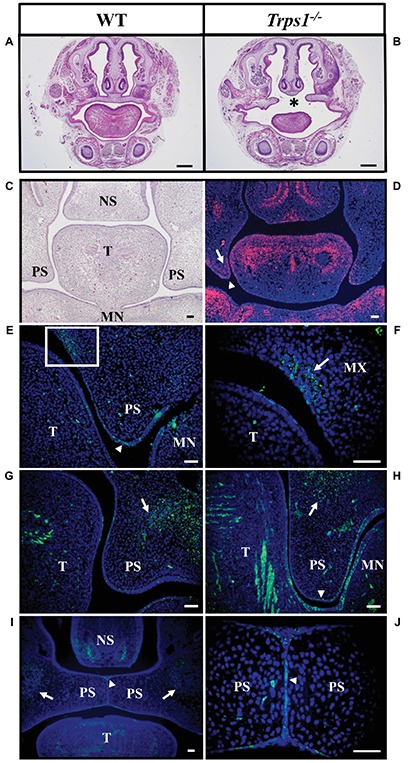
Cleft palate in *Trps1^−/−^* mice and the Trps1 expression pattern during normal mouse palatal development. **(A,B)** Representative images of H&E staining of frontal head sections of E18.5 WT control and *Trps1^−/−^* mice (129svev background). Asterisk indicates cleft palate in *Trps1^−/−^* mice. *Scale bar: 500 μm.* **(C)** H&E staining and **(D)** RNA *in situ* hybridization of WT frontal head sections of palatal shelves at E13.5. RNA *in situ* hybridization detected *Trps1* expression (red) in palatal shelf epithelium (arrowheads) and mesenchyme (arrows) at E13.5. *Trps1* expression is also detected in the tongue and nasal septum. **(E)** Immunoflourescent staining for Trps1 in E12.5 mice detected the Trps1 protein in the epithelium surrounding the palatal shelf (arrowhead). **(F)** Higher magnification of boxed area in **(E)** shows Trps1 presence in the maxillary process mesenchyme (arrow) and the adjacent epithelium. **(G)** Section of WT E13.5 anterior palate showing Trps1 in palatal shelf, maxillary mesenchyme (arrow) and tongue (T). **(H)** Section of WT E13.5 posterior palate showing Trps1 in maxillary mesenchyme (arrow), palatal shelf epithelium (arrowhead), and tongue (T). **(I)** At E14.5, Trps1 was detected in palatal shelf epithelium (arrowhead) and maxillary mesenchyme (arrows). **(J)** Higher magnification of image **(I)** at the palatal shelf fusion site shows Trps1 at MEE (arrowhead). *Scale bar: 50 μm. MN* – *mandible, MX* – *maxilla, NS* – *nasal septum, PS* – *palatal shelf, T* – *tongue*.

TRPS patients occasionally present with cleft palate (Morioka et al., 1999; Solc et al., 2017) and the same abnormality was noted in our previous studies of *Trps1^−/−^* mice (Kantaputra et al., 2008). Here, the skeletal staining revealed that the palatal shelves were still widely separated at E18.5 in *Trps1^−/−^* mice (Figure 2F). This was further confirmed by histological analysis (Figures 3A,B). Importantly, skeletal preparations and histological analyses detected cleft palate in all *Trps1^−/−^* mice analyzed. Thus, this abnormality shows complete penetrance and is independent of the genetic strain, as it was present in all *Trps1^−/−^* mice on C57BL/6J and 129svev background.

### Trps1 Is Expressed in Palatal Shelves Epithelium and Mesenchyme

Following the cleft palate phenotype in *Trps1^−/−^* mice, we focused on understanding the role of *Trps1* during palatogenesis. Previous studies have shown that during early embryogenesis (E11.5), *Trps1* is highly expressed in the first branchial arch mesenchyme (Kunath et al., 2002; Kantaputra et al., 2008). To delineate *Trps1* expression in the oral cavity, we used both RNA *in situ* hybridization and protein detection with immunostaining. Analysis of the *Trps1* expression pattern during the growth, elevation, and fusion of palatal shelves revealed *Trps1* expression in the mesenchyme of the presumptive vomer condensation and in the tips of the palatal shelves in the maxillary prominence. In addition, the tongue mesenchyme as well as mesenchyme of the mandibular process strongly expressed *Trps1* (Figures 3C,D). Specifically, at E12.5, Trps1 protein was detected in the mesenchyme and epithelium of palatal shelves, including the epithelium surrounding the palatal shelves at the future fusion sites (Figure 3E). Trps1 was also found in the nasal region of the palatal shelves (Figures 3E,F). At E13.5, the presence of Trps1 was more widespread in the palatal shelves, mandible, and tongue, especially in the posterior region of the oral cavity (Figures 3G,H). When the palatal shelves were in the adhesion/fusion stage at E14.5, Trps1 was detected in the maxillary mesenchyme and palatal shelf epithelium (Figure 3I). Trps1 expression was evident in the seam of medial edge epithelium (MEE) (Figure 3J), suggesting that Trps1 is involved in palatal shelf fusion.

### Trps1 Is Required for the Initiation of the Palatal Shelves Fusion

**Figure 4:**
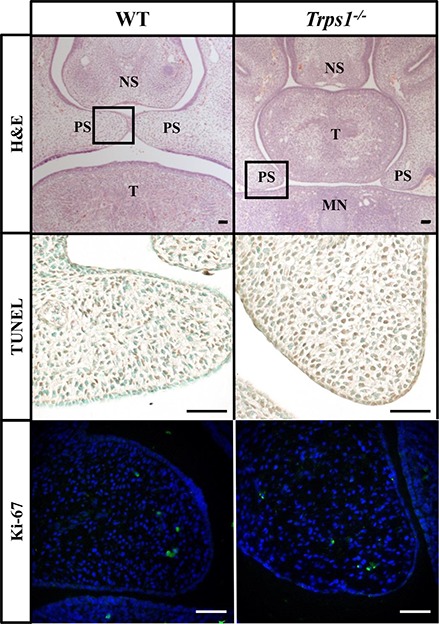
No difference in apoptosis or proliferation between E14.5 WT and *Trps1^−/−^* mice palatal shelves. Top: H&E staining of frontal head sections. Boxed areas indicate palatal shelves areas magnified on the images in the middle and bottom panels. Middle and bottom: TUNEL staining and immunofluorescent staining for Ki-67 of WT and *Trps1^−/−^* mice demonstrated no apparent difference in apoptosis or proliferation, respectively, between the two groups. *Scale bar: 50 μm. MN* – *mandible, NS* – *nasal septum, PS* – *palatal shelf, T* – *tongue*.

**Figure 5:**
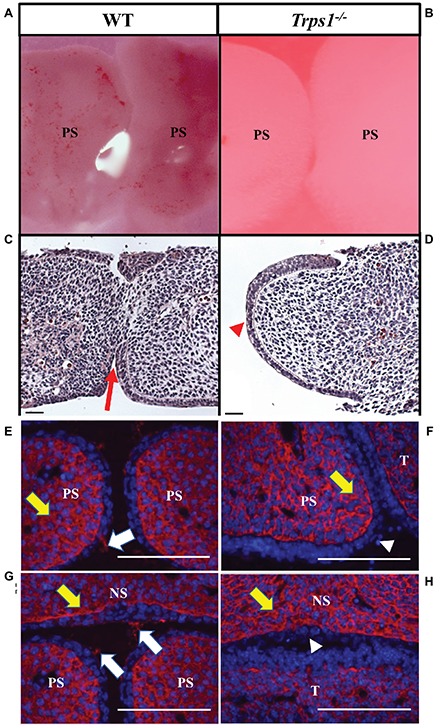
*Trps1* is required for the initiation of the palatal shelves fusion and expression of CSPG on palatal and nasal septum surfaces. **(A,B)** Stereoscope imaging and **(C,D)** H&E staining of palatal shelves isolated from E13.5 mice embryos and cultured *ex vivo* for 48 h. Of note, palatal shelves remained in contact with each other during *ex vivo* culture; however, *Trps1^−/−^* palatal shelves separated during histological processing, therefore only one palatal shelf is present on the image. Fusion of palatal shelves (red arrow) was initiated in all WT palatal shelves (*n* = 11) **(C)** while *Trps1^−/−^* palatal shelves **(D)** were unable to initiate fusion (red arrowhead, *n* = 11). **(E–H)** Immunohistochemistry for CSPG on fusion surfaces (white arrows) and mesenchyme (yellow arrows) of palatal shelves and nasal septum in E14.5 WT **(E,G)** and *Trps1^−/−^* **(F,H)** mice. CSPG was absent on the epithelial surface of *Trps1^−/−^* mice palatal shelves and nasal septum (white arrowheads), while the mesenchymal expression appeared unaffected. *Scale bar: 50 μm. NS* – *nasal septum, PS* – *palatal shelf, T* – *tongue.*

Given the expression of Trps1 in the palatal shelves and its previously reported effect on cell proliferation (Suemoto et al., 2007; Napierala et al., 2008; Wuelling et al., 2009), we compared proliferative and apoptotic activity by immunofluorescent detection of Ki-67 and TUNEL assay, respectively. These analyses did not detect any apparent differences in proliferation or apoptosis between WT and *Trps1^−/−^* E14.5 mice palatal shelves (Figure 4). Since Trps1 was detected specifically in the MEE, we focused our further analyses on the palatal shelves fusion process. To determine whether *Trps1* deficiency disrupts palatal shelves fusion, the *ex vivo* organ culture approach was used. Palatal shelves isolated from WT, *Trps^+/−^*, and *Trps1^−/−^* mice were placed in close contact with each other and remained in contact during the *ex vivo* culture (Figures 5A,B). After 48 h of *ex vivo* culture, fusion of 100% of WT and *Trps^+/−^* palatal shelves occurred (Figure 5C). However, none of the *Trps1^−/−^* palatal shelves pairs initiated the fusion process (Figure 5D). Given that palatal shelf adhesion is a prerequisite for the fusion, we examined the presence of the cell adhesion mediator chondroitin sulfate proteoglycan (CSPG) in palatal shelves (Gato et al., 2002). Immunohistochemistry confirmed the presence of CSPG on the fusion surface of the WT palatal shelves, but CSPG was undetectable on the surface of *Trps1^−/−^* palatal shelves and nasal septum (Figures 5E–H). Importantly, CSPG was detected in the palatal shelf mesenchyme of both WT and *Trps1^−/−^* mice, indicating that *Trps1* deficiency results in a cell type-specific loss of CSPG in the epithelium.

### Loss of Tgfβ3, β-Catenin, and Twist1 in *Trps1^−/−^* Palatal Shelves Epithelium

**Figure 6:**
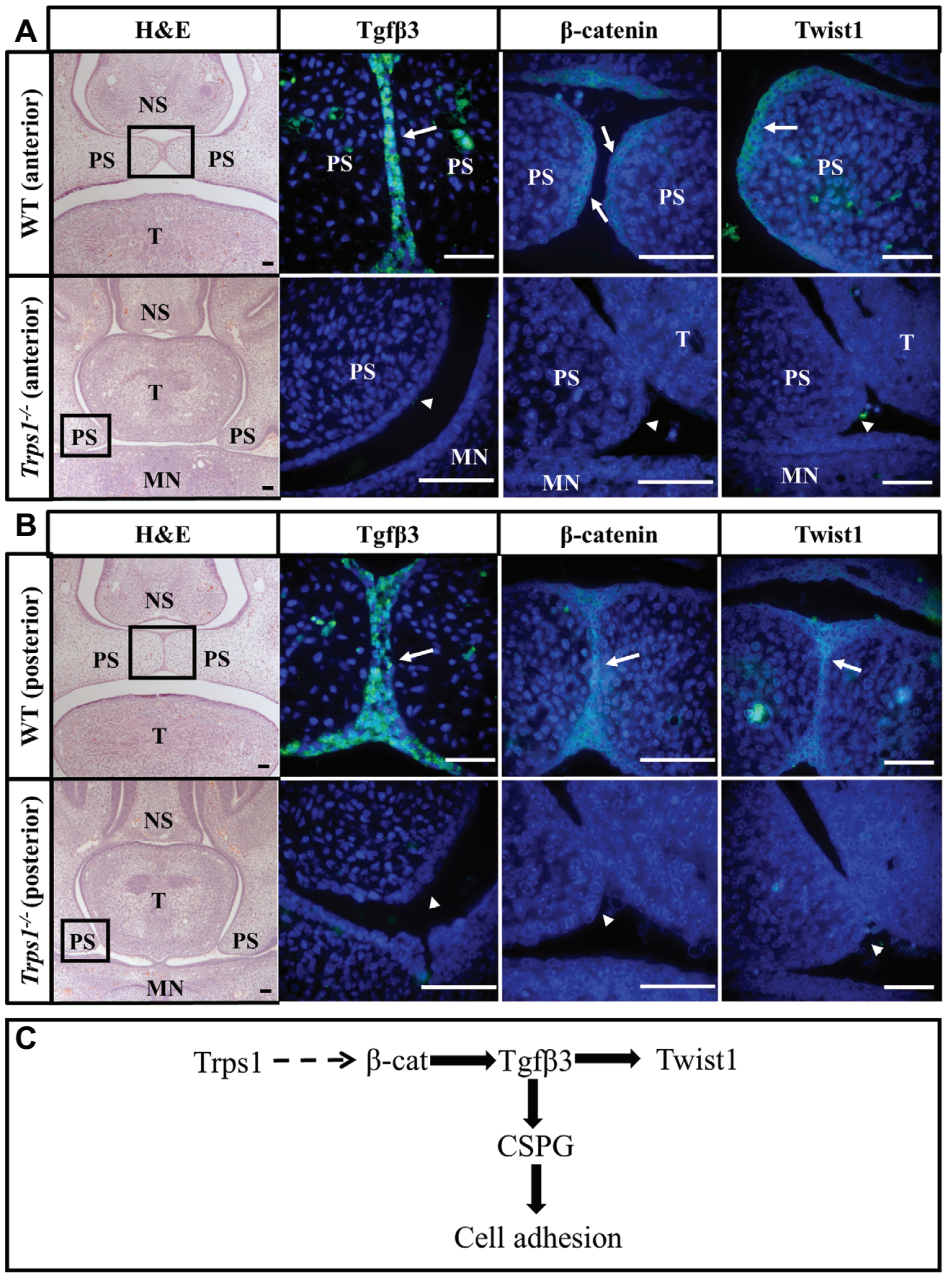
Loss of *Tgfβ3*, β-catenin, and *Twist1* expression in palatal shelf epithelium of *Trps1^−/−^* mice. H&E and immunofluorescent staining of E14.5 anterior **(A)** and posterior **(B)** palatal shelves in WT and *Trps1^−/−^* mice. Immunofluorescence images are of boxed areas marked on the H&E images. Tgfβ3 and Twist1 were present in MEE of both anterior and posterior WT palate but not in *Trps1^−/−^* palatal shelves. β-catenin was detected in the MEE of WT mice as well but was undetectable in *Trps1^−/−^* mice palatal shelves. **(C)** Diagram of a possible mechanism by which Trps1 regulates Tgfβ3, β-catenin, and Twist1 expression during palatal fusion. The direct relationship between Trps1 and β-catenin in palatal shelves is unclear. *Scale bar: 50 μm. MN* – *mandible, NS* – *nasal septum, PS* – *palatal shelf, T* – *tongue*.

It has been shown that the accumulation of CSPG at the fusion surfaces depends on the expression of transforming growth factor-beta 3 (*Tgfβ3*) in palatal shelf epithelium (Gato et al., 2002; Krauss, 2011). The association between *TGFβ3* mutation and cleft palate in both human and animal studies highlights the importance of this signaling molecule in palatogenesis (Proetzel et al., 1995; Carinci et al., 2007; Rienhoff et al., 2013). Thus, we investigated whether the loss of CSPG in *Trps1^−/−^* mice may be due to Tgfβ3 deficiency. As reported before, Tgfβ3 was highly and specifically expressed in the MEE of WT mice palate (Figures 6A,B). In contrast, Tgfβ3 protein was not detected in the *Trps1^−/−^* palatal shelves (Figures 6A,B). This suggests that CSPG deficiency on the fusion surface is a consequence of the loss of *Tgfβ3* expression in *Trps1^−/−^* palatal shelf epithelium.

The absence of Tgfβ3 in the *Trps1^−/−^* epithelium led us to investigate other proteins that are essential for palatal fusion and may be involved in Trps1 regulatory networks. We analyzed Twist1, which is the key EMT regulator required for palatal fusion and functions downstream of the Tgfβ3, and β-catenin, which has been implicated in both Trps1 and Tgfβ3 signaling (Yu et al., 2008; He et al., 2011; Fantauzzo and Christiano, 2012). Both Twist1 and β-catenin proteins were primarily present in the epithelium of the WT E14.5 palatal shelves, but their expression was undetectable in the *Trps1^−/−^* palatal shelves (Figures 6A,B). Therefore, these results suggest that Trps1 is required for the epithelial expression of several proteins critical for the palatal shelves fusion.

## Discussion

*TRPS1* involvement in the craniofacial development is evident by the characteristic facial phenotype of TRPS patients, but the exact role of *TRPS1* in this process has not been well studied. Here, to determine the role of *TRPS1* in craniofacial development, *Trps1^−/−^* mice were used to identify the craniofacial skeletal elements that rely on *Trps1* for normal development. We have delineated the expression pattern of *Trps1* during palatal development and determined that *Trps1* is required for the initiation of palatal shelf fusion and for the epithelial expression of several proteins critical for this process.

Our findings revealed that *Trps1* is involved in the development of the mandible and the cranial base. The hypoplasia of the zygomatic process of the maxillary bone and mandible in *Trps1^−/−^* mice is consistent with the TRPS clinical presentation in human patients (Ludecke et al., 2001; Griffiths et al., 2016). Other skeletal abnormalities demonstrated in patients with TRPS include a reduction in posterior cranial base (King and Frias, 1979). Here, we detected a hypoplasia of the anterior cranial base in *Trps1^−/−^* mice with an underdeveloped basisphenoid and the complete absence of presphenoid ossification at E18.5, which has not been described before. Further examination of the mandible also revealed cartilaginous hypoplasia at the condylar and angular processes in *Trps1^−/−^* mice. This is consistent with the previous findings identifying *Trps1* as a regulator of chondrocyte proliferation and differentiation in appendicular skeleton and TMJ (Suemoto et al., 2007; Napierala et al., 2008; Wuelling et al., 2009; Michikami et al., 2012). Finally, delayed tooth eruption, which is frequently observed in TRPS patients, was also found in the E18.5 *Trps1^−/−^* mice. The craniofacial characteristics of *Trps1* homozygous mutant mice are highly comparable to the phenotype of patients with *TRPS1* haploinsufficiency but with increased severity, indicating the dose-dependent effect of *Trps1* deficiency. Of note, despite the hypoplasia of the facial and cranial base elements, cranial vault development is mostly unaffected (Figure 2J).

We demonstrated that *Trps1* is expressed in multiple sites prior to palate formation, including the mesenchyme of maxilla, mandible, tongue, and nasal septum. This suggests that *Trps1* deficiency can disrupt multiple steps of palatogenesis. For example, the expression of *Trps1* in palatal shelf mesenchyme opens up the possibility of *Trps1* playing a role in palatal shelf elevation as this process is thought to be guided by internal forces (Li et al., 2017). This possibility is supported by the fact that at E14.5, WT palatal shelves have already elevated and begun to approximate each other while the *Trps1^−/−^* palatal shelves were still oriented vertically (Figures 6A,B). The expression of *Trps1* in the tongue with a striated pattern, indicating expression in glossal muscles, suggests that it could also play a role in tongue descent (Figure 3G). In some craniofacial disorders such as Pierre Robin sequence, the cleft palate phenotypes are caused by a failure of tongue descent associated with micrognathic mandible (Levi et al., 2011). Although the small mandible of *Trps1^−/−^* mice and TRPS patients suggests that this might be the case in TRPS, histological analyses of E18.5 in *Trps1^−/−^* mice revealed palatal shelves elevated above the tongue (Figure 3B). Hence, it is unlikely that failure of tongue descent is the main underlying cause of cleft palate in *Trps1* deficiency.

The expression of *Trps1* in both palatal shelf mesenchyme and MEE (Figures 3E–J) raises the possibility that the cleft palate phenotype in *Trps1^−/−^* mice could arise from a combination of palatal shelf outgrowth disruption and palatal shelf fusion failure. Although we detected delayed elevation of palatal shelves in *Trps1^−/−^* mice, we did not detect differences in cell proliferation or apoptosis between WT and *Trps1^−/−^* mice (Figure 4). Therefore, we focused this study on the processes of palatal shelf fusion. In the *ex vivo* organ culture experiments, we uncovered that *Trps1^−/−^* palatal shelves do not even initiate the fusion process. This failure is most likely caused by an inability of the *Trps1^−/−^* palatal shelves to adhere to each other due to the absence of CSPG, as was demonstrated by immunostaining. Interestingly, only epithelial expression of CSPG depends on *Trps1*, while expression of CSPG in mesenchyme is unaffected (Figures 5E–H). This indicates that CSPG expression is regulated by *Trps1 via* a cell type-specific mechanism. Our finding that Tgfβ3 was absent in *Trps1^−/−^* palatal epithelium strongly suggests that CSPG deficiency is a consequence of the loss of Tgfβ3 (Figure 6C). Trps1 has been shown to interact with the Tgfβ and canonical Wnt signaling pathways in kidney and hair follicles, respectively (Gai et al., 2009; Fantauzzo and Christiano, 2012). However, the mechanism by which *Trps1* deficiency decreases the expression of Tgfβ3, β-catenin, and Twist1 in the MEE is unknown. Although most of the studies demonstrate that Trps1 is a transcriptional repressor (Malik et al., 2001; Napierala et al., 2008), it has been determined that, at least in the context of hair follicle progenitors, Trps1 can act as a transcriptional activator (Fantauzzo and Christiano, 2012). This opens the possibility that expression of *Tgfβ3, β-catenin*, or *Twist1* might be directly activated by Trps1 in palatal shelf epithelium.

The signaling pathways of Tgfβ3, β-catenin, and Twist1 are all interconnected during palatal development (Figure 6C). Studies have demonstrated that β-catenin is necessary for Tgfβ3 regulation in palatal shelf epithelium while Tgfβ3 activates *Twist1* expression during the early stages of palatal fusion (Yu et al., 2008; He et al., 2011). Tgfβ3 and Twist1 are essential for palatal fusion due to their role in *E-cadherin* suppression, which is the hallmark of EMT (Yu et al., 2009; Ke et al., 2015). Interestingly, *Trps1* is an inhibitor of EMT in tumor and liver cells and Trps1 levels were positively correlated with E-cadherin (Su et al., 2014; Zhe et al., 2015; Huang et al., 2016). While these data from malignant tissues support the role of *Trps1* in EMT, the developmental functions of *Trps1* may also stem from its regulation of additional genes such as *Tgfβ3* and *β-catenin* to promote cell adhesion or EMT during palatogenesis.

In summary, our study uncovered the importance of *Trps1* in craniofacial development and, specifically, palatogenesis. Further investigations will address the molecular mechanisms by which *Trps1* regulates expression of genes required for the initiation of the palatal shelf fusion to advance our understanding of the molecular mechanisms of cleft palate in cases of *Trps1* deficiency. Although here we examined the processes of palatal shelf adhesion/fusion, it is also important to explore the role of *Trps1* in other stages of palatogenesis, including palatal shelf outgrowth and elevation, tongue descent, apoptosis or EMT, as those might be also affected by a *Trps1* deficiency.

## Ethics Statement

This study was carried out in accordance with the recommendations of University of Pittsburgh Research Conduct and Compliance Office. The protocol was approved by the University of Pittsburgh Institutional Biosafety Office and by Institutional Animal Care and Use Committee.

## Author Contributions

KYC, BL, HS-R, and DN contributed to the conception and design of the study. KYC, BK, DM, and DN performed data acquisition. KYC, DN, and HS-R contributed to data analysis and interpretation. KYC wrote the first draft of the manuscript. All authors contributed to manuscript revision, read and approved the submitted version.

### Conflict of Interest Statement

The authors declare that the research was conducted in the absence of any commercial or financial relationships that could be construed as a potential conflict of interest.
